# AGEISM IN THE LABOR MARKET: LEARNING AND EDUCATION FOR OLDER ADULT JOB SEEKERS

**DOI:** 10.1093/geroni/igad104.2321

**Published:** 2023-12-21

**Authors:** Bora Jin, Lisa Baumgartner

**Affiliations:** Winston Salem State University, Winston-Salem, North Carolina, United States; Texas State University, San Marcos, Texas, United States

## Abstract

Despite older workers’ value to an organization and no differences in job performance or productivity from younger workers (Rego et al., 2018), older job seekers encounter ageism in employment during their job search (Zaniboni et al., 2019). Ageism in employment and the workplace have detrimental effects on older job seekers, organizations, and society. Therefore, this conceptual paper explores the financial, emotional, psychological, and physical factors that should be considered when supporting older job seekers in navigating reeducation and learning to obtain employment. We collected and reviewed extant literature on this topic by consulting the following databases: EBSCOhost, PsycINFO, Google Scholar, and PubMed and using search terms such as “older adult,” “workplace,” “ageism,” “employment.” Ageist practices and stereotypes can occur at any stage of the employment process, including application, interview, hiring, training, job duties, benefits, downsizing, or termination. Older job seekers or employees may experience age discrimination in the workplace directly (e.g., if employers consider candidates cannot project the youthful image that the business desires, they may decline to offer the position) or indirectly (employers reject older applicants for a job because of their overqualification). We provide practical implications for older job seekers by exploring workplace learning strategies for older adults, including peer mentoring, intergenerational learning (Arthanat, 2019), proactive personality, and effective job search activity (Zacher, 2013). In addition, we discuss how older workers need to see the relevance of tasks to be individually motivated to learn and explore the need for personalized learning programs (Seo et al., 2019).

